# Scheduling Jobs and a Variable Maintenance on a Single Machine with Common Due-Date Assignment

**DOI:** 10.1155/2014/748905

**Published:** 2014-07-22

**Authors:** Long Wan

**Affiliations:** School of Information Technology, Jiangxi University of Finance and Economics, Nanchang 330013, China

## Abstract

We investigate a common due-date assignment scheduling problem with a variable maintenance on a single machine. The goal is to minimize the total earliness, tardiness, and due-date cost. We derive some properties on an optimal solution for our problem. For a special case with identical jobs we propose an optimal polynomial time algorithm followed by a numerical example.

## 1. Introduction

Recently, as a competitive strategy to provide high quality service for customer demand, just-in-time (JIT) production has received considerable attention from the manufacturing enterprises [1]. In JIT production, jobs should be completed as close as possible to their due-dates. A job which is completed earlier or later than its due-date will incur penalty. Thus, both earliness and tardiness are discouraged in JIT production. Motivated by the JIT production, in the last two decades scheduling problem with due-date assignment has been extensively investigated. For the related surveys, we refer the readers to Cheng and Gupta [2], Baker and Scudder [3], and Gordon et al. [4]. For some recent related models on the due-date assignment scheduling, see Xu et al. [5], Gerstl and Mosheiov [6], Yin et al. [7], and Janiak et al. [8].

On the other hand, to prevent production disruption caused by machine breakdown, machine maintenance needs to be performed to perserve production efficiency. Since 1996, researchers begun to take maintenance into consideration in scheduling (see Lee [9], Lee and Chen [10], Kubzin and Strusevich [11], Mosheiov and Sidney [12], and Zhao and Tang [13]). For a maintenance (which is optional or mandatory), we usually use two parameters to define it. One is its starting time and the other is its duration. In some papers, the occupied period by maintenance was also called nonavailable interval. For the recent related survey, we refer the readers to Ma et al. [14].

In the papers by Kubzin and Strusevich [11] and Mosheiov and Sidney [12], they considered a more realistic case on the duration of maintenance. In their models, they assume the duration of maintenance is variable; that is, the duration of maintenance depends on its starting time in that the later maintenance is performed, the longer time is needed to perform the maintenance. Such maintenance can be called a variable maintenance or a deteriorating maintenance.

Another popular topic in recent years is that of scheduling with simultaneous considerations of due-date assignment and maintenance. Mosheiov and Oron [15] studied a single-machine scheduling problem jointly with rate-modifying activity and common due-date assignment considerations to minimize the total of earliness, tardiness, and due-date costs. X. Y. Wang and M. Z. Wang [16] addressed a single-machine slack due-date scheduling with a rate-modifying activity for minimizing the objective function which contains the total earliness, tardiness, and the common slack time costs. Yang et al. [17] investigated single-machine common due-date assignment and scheduling problems with an aging effect under a deteriorating maintenance consideration simultaneously. Yin et al. [18] considered a common due-date assignment and scheduling problem with a rate-modifying activity to minimize the due-date, earliness, tardiness, holding, and batch delivery cost.

In this paper we introduce a new scheduling model which combines the due-date assignment and the machine maintenance. We assume that the duration of maintenance is variable and the maintenance must be started prior to a given deadline.

As a practical example for the proposed model, we may consider the steel-making process in the steel plant [19]. In the steel-making process, a charge, that is, a concurrent smelting in the same converter, is regarded as a “job.” A refining furnace is used to refine the charges. Naturally, the refining furnace is regarded as “the machine.” In the refining process, there will be some garbage. Before a given deadline, we must clear the garbage to maintain the production efficiency and thus the clearing operation can be regarded a maintenance.

In the second section we provide the notation and formulation on our model. The third section derives some important properties on an optimal solution. In Section 4, we propose an optimal polynomial time algorithm for a special case with identical jobs, followed by a numerical example. Concluding remarks are discussed in the last section.

## 2. Problem Statement

Our problem can be described as follows. There are *n* independent jobs *J*_1_, *J*_2_,…, *J*_*n*_ to be nonpreemptively processed on a single machine, all of which are available at time zero. A mandatory maintenance must be started before a given deadline on the machine and the duration of maintenance depends on its starting time; that is, the duration is a nonnegative and nondecreasing function of the starting time. Let *p*_*j*_ and *d*_*j*_ denote the processing time and the due-date of job *J*_*j*_, *j* = 1,2,…, *n*, respectively. For a given schedule, we use *C*_*j*_ to denote the completion time of job *J*_*j*_, *j* = 1,2,…, *n*. We define the earliness and tardiness of job *J*_*j*_ as *E*_*j*_ = max⁡{*d*_*j*_ − *C*_*j*_, 0} and *T*_*j*_ = max⁡{*C*_*j*_ − *d*_*j*_, 0}, *j* = 1,2,…, *n*, respectively. The unit earliness and tardiness penalties are denoted by *α* (>0) and *β* (>0), respectively. In the case of a common due-date (i.e., *d*_*j*_ = *d*, which is a decision variable), we denote the penalty per unit time of delaying the due-date by *γ* (>0). Furthermore, we denote the given deadline of maintenance with *s*_*g*_ and the duration of maintenance with *l*. Then according to our previous assumption, we can denote *l* = *f*(*s*), where *s* (≤*s*_*g*_) is the starting time of maintenance and *f* is a nonnegative and nondecreasing function. The goal is to find an optimal sequence of all the jobs, the common due-date, and the staring time of maintenance such that the objective ∑_*j*=1_^*n*^(*αE*_*j*_ + *βT*_*j*_) + *γd* is minimized. Following the three-field notation proposed by Graham et al. [20], we denote our problem as 1, VM||∑_*j*=1_^*n*^(*αE*_*j*_ + *βT*_*j*_) + *γd*, where VM in the first field stands for a variable maintenance.

## 3. The Properties on an Optimal Solution

The classical due-date assignment scheduling problem (without maintenance) was introduced by Panwalkar et al. [21]. In their model, in addition to the traditional job sequencing decisions, the common due-date is a decision variable. Both earliness and tardiness incur penalty cost. The goal is to find an optimal sequence of the jobs and the due-date that minimizes the total earliness, tardiness, and due-date cost. By using the small perturbations technique, Panwalkar et al. [21] proposed a polynomial time algorithm.

In order to solve our problem, we first derive some properties on an optimal solution. We also use the small perturbations technique.

**Lemma 1 .**

There exists an optimal solution in which the schedule starts at time zero and contains no idle time among the jobs, and the maintenance is scheduled between the two consecutive jobs without idle time.

**Proof**

First we show that there is no idle time between the jobs.

**Figure 1:**
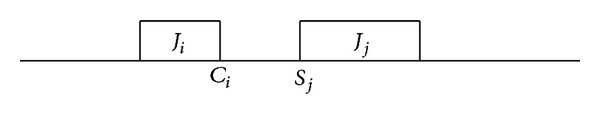
Idle time between jobs *J*_*i*_ and *J*_*j*_.

Assume that there exists idle time between the jobs *J*_*i*_ and *J*_*j*_, as shown in Figure 1, where *C*_*i*_ denotes the completion time of job *J*_*i*_ and *S*_*j*_ denotes the starting time of job *J*_*j*_. Clearly we have *C*_*i*_ < *S*_*j*_. If *d* < *C*_*i*_, we move job *J*_*j*_ by (*S*_*j*_ − *C*_*i*_) units of time to the left without increasing the objective value. If *d* > *S*_*j*_, we may move job *J*_*i*_ by (*S*_*j*_ − *C*_*i*_) units of time to the right without increasing the objective value. If *C*_*i*_ ≤ *d* ≤ *S*_*j*_, we may move job *J*_*i*_ to the right and job *J*_*j*_ to the left such that job *J*_*i*_ just finishes at time *d* and job *J*_*j*_ starts at time *d* without increasing the objective value. In the end, by clearing the idle time between the jobs we always obtain a better solution.

Next, we show that the maintenance is scheduled between the two consecutive jobs without idle time.

**Figure 2:**
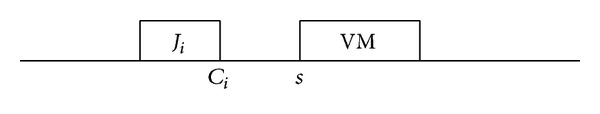
Idle time between job *J*_*i*_ and the maintenance VM.

Assume that there exists idle time between the job *J*_*i*_ and the maintenance, as shown in Figure 2, where *J*_*i*_ is scheduled before the maintenance and *s* denotes the starting time of the maintenance. Clearly, we have *C*_*i*_ < *s*. If *d* < *C*_*i*_, we move the maintenance by (*s* − *C*_*i*_) units of time to the left without increasing the objective value. If *d* > *s*, we may move job *J*_*i*_ by (*s* − *C*_*i*_) units of time to the right without increasing the objective value. If *C*_*i*_ ≤ *d* ≤ *s*, we may move job *J*_*i*_ to the right and the maintenance to the left such that job *J*_*i*_ finishes at time *d* and the maintenance starts at time *d* without increasing the objective value.

**Figure 3:**
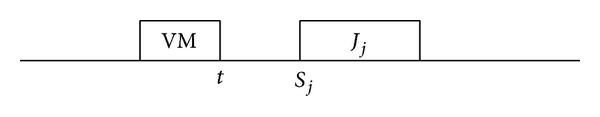
Idle time between the maintenance VM and job *J*_*j*_.

Assume that there exists idle time between the maintenance and the job *J*_*j*_, also shown in Figure 3, where *J*_*j*_ is scheduled after the maintenance and *t* denotes the finishing time of the maintenance.

Clearly, we know *t* < *S*_*j*_. If *d* < *t*, we move job *J*_*j*_ by (*S*_*j*_ − *t*) units of time to the left without increasing the objective value. If *d* > *S*_*j*_, we may delay the starting time of maintenance such that the maintenance finishes at time *S*_*j*_. If *t* ≤ *d* ≤ *S*_*j*_, we may delay the starting time of maintenance and move job *J*_*j*_ to the left such that the maintenance finishes at time *d* and job *J*_*j*_ starts at time *d*.

By the above analysis, we can treat all the jobs and the maintenance as a consecutive whole without idle time.

Finally, we show that the schedule starts at time zero. Assume that there exists a solution which does not start at time zero. Then we move the whole to the left by some times to assure that the new schedule starts at time zero and reset a smaller common due-date than the original due-date to obtain a new solution, which does not increase the objective value.

With the above argument, we conclude Lemma 1 holds.

**Lemma 2 .**

The optimal common due-date is the completion time of the job in position *m*, where *m* = ⌈(*nβ* − *γ*)/(*α* + *β*)⌉.

**Proof**

First we show that in an optimal solution the common due-date *d* is the completion time of some job. We distinguish two cases.

*Case 1*. Consider a solution with *C*_*i*_ < *d* < *C*_*i*+1_, where *i* denotes the job scheduled in the *i*th location. Let *Z* be the corresponding objective value. Define *x* = *d* − *C*_*i*_ and *y* = *C*_*i*+1_ − *d*. Let *Z*_1_ and *Z*_2_ be the objective value for *d* = *C*_*i*_ and *d* = *C*_*i*+1_. Then

(1) Z1=Z+β(n−i)x−αxi−γx=Z+x(β(n−i)−αi−γ),Z2=Z−β(n−i)y+αiy+γy=Z−y(β(n−i)−αi−γ).

Thus, we have *Z*_1_ ≤ *Z* if *β*(*n* − *i*) − *αi* − *γ* ≤ 0 and *Z*_2_ ≤ *Z* otherwise. This implies that an optimal solution exists in which *d* is equal to the completion time of some job.

**Figure 4:**
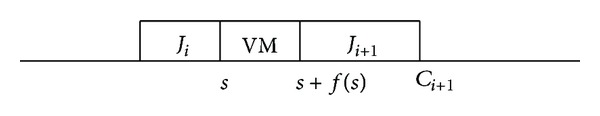
The case *s* ≤ *d* ≤ *s* + *f*(*s*).

*Case 2*. Consider a solution with *s* ≤ *d* ≤ *s* + *f*(*s*), where *s* denotes the starting time of maintenance and *f*(*s*) denotes the duration of maintenance. Similar to Case 1, using the small perturbations technique we can show that the objective value can reduce by resetting *d* = *s* or *d* = *s* + *f*(*s*). Since the case *d* = *s* is shown in Case 1, thus we only need to consider the case *d* = *s* + *f*(*s*). Let *Z* be the corresponding objective value with *d* = *s* + *f*(*s*) and *Z*_1_ and *Z*_2_ the corresponding objective values for *d* = *C*_*i*_ and *d* = *C*_*i*+1_, where the maintenance is scheduled between the job *J*_*i*_ and the job *J*_*i*+1_; that is, *s* = *C*_*i*_ and *s* + *f*(*s*) = *C*_*i*+1_ − *p*_*i*+1_, as shown in Figure 4.

Then

(2) $$\begin{array}{c} Z_{1}=Z+f\left(s\right)\left(\beta \left(n-i\right)-\alpha i-\gamma \right),\\ Z_{2}=Z-p_{i+1}\left(\beta \left(n-i\right)-\alpha i-\gamma \right). \end{array}$$

Thus, we have *Z*_1_ ≤ *Z* if *β*(*n* − *i*) − *αi* − *γ* ≤ 0 and *Z*_2_ ≤ *Z* otherwise.

With the above discussion, we conclude that in an optimal solution the optimal common due-date *d* is the completion time of some job.

Now, we assume that the common due-date *d* is the completion time of job in the *m*th location; that is, *d* = *C*_*m*_. To prove that *m* = ⌈(*βn* − *γ*)/(*α* + *β*)⌉, let *Z* be the objective value of optimal solution. Applying *Z*_1_ and *Z*_2_ to the situation that *x* = *d* − *C*_*m*−1_ and *y* = *C*_*m*+1_ − *d*, respectively, we conclude that

(3) $$\begin{array}{c} \beta \left(n-m+1\right)-\alpha \left(m-1\right)-\gamma \geq 0,\\ \beta \left(n-m\right)-\alpha m-\gamma \leq 0. \end{array}$$

Thus, we have *m* = ⌈(*nβ* − *γ*)/(*α* + *β*)⌉.

**Lemma 3 .**

In an optimal solution, the maintenance is scheduled either at time 0, or after the common due-date.

**Proof**

**Figure 5:**
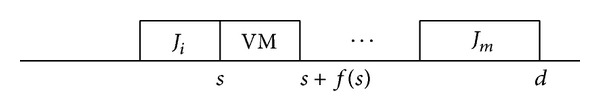
The maintenance starts at time *s* (>0).

Suppose that there exists a solution in which the maintenance starts at time *s*, where *s* > 0 and is scheduled before the common due-date *d*. Then the maintenance occupies the time interval  [*s*, *s* + *f*(*s*)] with *s* ≤ *s*_*g*_ and *s* + *f*(*s*) ≤ *d*. Furthermore, we assume that the job *J*_*i*_ is just prior to the maintenance and the completion time of job *J*_*m*_ is equal to the due-date, as shown in Figure 5.

**Figure 6:**
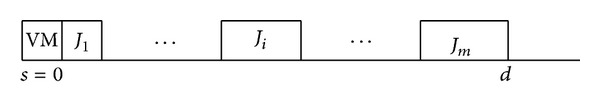
The maintenance starts at time 0.

Now we construct a new solution as follows. Starting the maintenance at time zero and scheduling all the jobs according to their original order just after the maintenance. Setting the common due-date to the new completion time of job *J*_*m*_. As shown in Figure 6, then we have the following.

- The duration of the maintenance decreases as it starts earlier.
- The earliness of jobs *J*_*i*_ and its predecessors are reduced.
- The common due-date *d* is reduced.

The above (i), (ii), and (iii) imply that the total earliness cost of job *J*_*i*_ and its predecessors and the due-date cost are reduced, and the earliness and tardiness cost of other jobs remain unchanged. Thus, we conclude that the maintenance should be scheduled either at time 0, or after the due-date.

## 4. A Special Case 1, VM|*p*_*j*_  =  *p*|∑_*j*_(*αE*_*j*_ + *βT*_*j*_) + *γd*

In this section we consider a special case for our problem. We assume all the jobs are identical; that is, *p*_*j*_ = *p*. Next, we propose a polynomial time algorithm for this special case based on the previous properties on an optimal solution.

Recall that the due-date is the completion time of job in the *m*th location, where *m* = ⌈(*βn* − *γ*)/(*α* + *β*)⌉. Because the jobs are identical jobs, by Lemma 3 we claim that the maintenance must be started at time 0 if *d* = *mp* > *s*_*g*_ and the maintenance is started after the due-date otherwise. Then there are at most *n* − *m* + 1 choices for the starting time of the maintenance.

With the above analysis, we propose our algorithm as follows.

*Algorithm H.*

**Step 1 .**

If *d* = *mp* > *s*_*g*_, where *m* = ⌈(*βn* − *γ*)/(*α* + *β*)⌉, construct schedule *π* = (VM, *J*_1_, *J*_2_,…, *J*_*n*_). Output it as our solution by setting the due-date as the completion time of job *J*_*m*_ and stop. Otherwise go to Step 2.

**Step 2 .**

Compute *k* such that *pk* ≤ *s*_*g*_ ≤ *p*(*k* + 1). Construct a series of schedules *π* ^0^ = (VM, *J*_1_, *J*_2_,…, *J*_*n*_), *π* ^*i*^ = (*J*_1_, *J*_2_,…, *J*_*i*_, VM, *J*_*i*+1_,…, *J*_*n*_), *i* = *m*, *m* + 1,…, *k*.

**Step 3 .**

Output the schedule with the minimal objective value from all the constructed schedules *π* ^0^, *π* ^*i*^, *i* = *m*, *m* + 1,…, *k*, and denote it as *π*, where *Z*(*π*) = *Min*⁡_*i*=0,*m*,*m*+1,…,*k*_ *Z*(*π* ^*i*^).

From the properties on an optimal solution as shown in Lemmas 1, 2, and 3, we conclude that Algorithm H is correct since all the possible cases are tried and we select the best one. For a given schedule, the computation of objective value requires *O*(*n*) time. There are at most *n* + 1 schedules to be considered; thus the total running time is *O*(*n* ^2^). Finally we obtain the following.

**Theorem 4 .**

The 1, *VM*|*p*_*j*_ = *p*|∑_*j*_(*αE*_*j*_ + *βT*_*j*_) + *γd* problem can be solved in *O*(*n* ^2^)  time.

*A Numerical Example.* To illustrate Algorithm H, a solution of an instance of 10 jobs is demonstrated in the following.

The job processing times are identical with *p*_*j*_ = 3, *j* = 1,2,…, 10. The deadline of maintenance *s*_*g*_ is equal to 25 and the duration of maintenance *l* is equal to 2 + *s*/3, where *s* (≤*s*_*g*_), as a decision variable, is the starting time of maintenance. The penalty parameters are as follows: *α* = 2, *β* = 3, and *γ* = 4.

Applying Algorithm H, we first compute the parameters as follows: *m* = ⌈(*βn* − *γ*)/(*α* + *β*)⌉ = ⌈(3 × 10 − 4)/(2 + 3)⌉ = 6, *d* = *mp* = 6 × 3 = 18, and *k* = 8 with 3 × *k* ≤ *s*_*g*_ ≤ 3 × (*k* + 1). Because 16 < 25, that is, *d* < *s*_*g*_, we construct a series of schedules as follows:

(4) π0=(VM,J1,J2,…,J10),π6=(J1,J2,…,J6,VM,J7,J8,…,J10),π7=(J1,J2,…,J6,J7,VM,J8,…,J10),π8=(J1,J2,…,J6,J7,J8,VM,J9,J10).

Their corresponding objective values are *Z*(*π* ^0^) = 260, *Z*(*π* ^6^) = 348, *Z*(*π* ^7^) = 324, and *Z*(*π* ^8^) = 300.

When comparing the costs in *π* ^0^, *π* ^6^, *π* ^7^, and *π* ^8^, we conclude that the global optimum is obtained in *π* ^0^, the maintenance starts at time zero, the common due-date *d* is equal to 20, six jobs are early, and four jobs are tardy. The total cost is *Z*(*π* ^0^) = 260.

## 5. Concluding Remarks

In this paper we consider the common due-date assignment scheduling problem with a variable maintenance on a single machine. The goal is to minimize the total earliness, tardiness, and due-date cost. We derive some properties on an optimal solution for our problem. For a special case with identical jobs we propose an optimal polynomial time algorithm running in *O*(*n* ^2^) time.

For the general case with nonuniform processing times of jobs, whether problem is NP-hard or not is open and deserves the further research.
